# Association Between Dietary Diversity and Subjective Cognitive Decline in the Middle-Aged and Elderly Chinese Population: A Cross-Sectional Study

**DOI:** 10.3390/nu16213603

**Published:** 2024-10-23

**Authors:** Minjie Gao, Jing Wang, Yue Qiu, Yanan Chen, Qiancheng Cao, Yiru Pan, Yifei Cao, Shufen Han, Xiao Yan, Xianrong Xu, Xuexian Fang, Fuzhi Lian

**Affiliations:** 1 Department of Nutrition and Toxicology, School of Public Health, Hangzhou Normal University, Hangzhou 311121, Chinayfcao@hznu.edu.cn (Y.C.); sfhan@hznu.edu.cn (S.H.); yx1986@hznu.edu.cn (X.Y.); xuxianrong@bjmu.edu.cn (X.X.); xfang@hznu.edu.cn (X.F.); 2 Xinchang Center for Disease Control and Prevention, Xinchang 312500, China; 3 Engineering Research Center of Mobile Health Management System, Ministry of Education, Hangzhou 311121, China

**Keywords:** subjective cognitive decline, dietary diversity, SCD-Q9, Dietary Quality Questionnaire

## Abstract

Background: This cross-sectional study aimed to examine the association between dietary diversity and risk of subjective cognitive decline (SCD), a precursor of dementia, in middle-aged and elderly Chinese populations residing in eastern China. Methods: Participants aged ≥ 45 years were recruited from a community in an eastern Chinese city after excluding potential objective cognitive impairment using the Mini-Cognitive Assessment Instrument (Mini-Cog). SCD was assessed using the Subjective Cognitive Decline Questionnaire-9 (SCD-Q9). Dietary data were collected using the Dietary Quality Questionnaire (DQQ), and the Food Group Diversity Score (FGDS) and the Consumed All Five Recommended Food Score (All-5) were calculated as indicators of dietary diversity. Odds ratios (*ORs*) and 95% confidence intervals (*CIs*) were computed to evaluate the associations of FGDS and All-5 scores with SCD after adjusting for age, sex, socioeconomic status, lifestyle factors, and health status. Results: Among the 871 participants, 358 (41.1%) were classified as having SCD. Compared with participants with the highest FGDS (≥8) and those with the highest All-5 score (5), those with the lowest FGDS (≤4) and the lowest All-5 score (≤3) exhibited 85% (*OR* = 1.85; *95% CI*: 1.10–3.13; *p* = 0.02) and 90% (*OR* = 1.90; *95% CI*: 1.21–2.97; *p* < 0.01) higher risk of SCD, respectively, after adjusting for all covariates. Fruits were the only food group among the All-5 components that demonstrated a significant association with SCD risk. Conclusions: Poor dietary diversity was associated with an elevated risk of SCD in middle-aged and older adults, and fruits were the food group with the most substantial effect.

## 1. Introduction

With the rapid aging of the world population, dementia has become a major disease, affecting the health of the elderly and posing a huge burden on the family and society [1]. Owing to the lack of effective treatments, preventive approaches that focus on early development of the disease are critical. Subjective cognitive decline (SCD), a state of self-perceived cognitive decline without detectable objective neuropsychological dysfunction, has been identified as a predementia condition preceding the development of mild cognitive impairment (MCI) [2,3]. As a common complaint among older populations, the prevalence of SCD ranges from approximately 10% among individuals aged 45 to 64 years [4] to more than 50% among those aged 70 years and older [2]. Studies have reported an approximately doubled risk of progressing to MCI and dementia among elderly individuals with SCD [5,6,7]. SCD may reflect the underlying development of dementia-associated brain pathologies, such as white matter degeneration, gray matter atrophy, and amyloid deposition [3,8]. SCD may occur decades before the onset of measurable cognitive impairment [9], making it an ideal window for the early prevention of cognitive impairment.

Diet is an important lifestyle factor that can modify the risk of cognitive impairment and dementia [10,11]. Studies have demonstrated that lower energy consumption [12], higher dietary intake of carotenoids [13] and flavonoids [14], and better adherence to healthy dietary patterns [15,16,17] are associated with a lower SCD risk, highlighting the importance of high-quality diets in the prevention of SCD. Dietary diversity, defined as the number of different foods or food groups consumed within a specific timeframe, is a key element of high-quality diets [18] and has been recommended by most dietary guidelines worldwide [18,19,20]. Better dietary diversity has been associated with a lower risk of cognitive decline or impairment [21,22,23,24,25]; however, its relationship with SCD has not been extensively investigated [26].

The dietary diversity score is usually calculated based on data from dietary recall and food frequency questionnaires, which are expensive, complex, and time-consuming. Recently, a low-burden diet quality questionnaire (DQQ) was developed as a food group-based tool for rapid dietary quality assessment at the population level [27,28]. Currently, only a few studies have linked DQQ-derived indicators of dietary diversity to health outcomes, such as psychological stress in adults [29] and obesity in children and adolescents [30]. In this preliminary study, we tested the hypothesis that dietary diversity indicators derived from the DQQ were negatively associated with SCD risk, and further examined the food group that was most significantly related to SCD in middle-aged and elderly community residents in eastern China.

## 2. Materials and Methods

### 2.1. Study Design and Participants

This cross-sectional study was conducted in Hangzhou, Zhejiang Province, China, between May 2023 and July 2023. The participants were community-dwelling residents aged 45 years and older who visited a local community health service center for routine health examinations. We excluded those who had been diagnosed with AD, Parkinson’s disease, or other forms of dementia and those with a history of severe mental disorders, stroke, or brain injury. Eligible participants were interviewed by trained investigators. A total of 969 residents were recruited for the study. After excluding 23 individuals with missing information and 75 with potential cognitive impairment, 871 participants were included in the final analysis. The study was conducted in accordance with the guidelines of the Declaration of Helsinki and approved by the Ethics Committee of Hangzhou Normal University. Written informed consent was obtained from all participants.

### 2.2. Cognitive Function Assessment

The Chinese version of the Mini-Cognitive Assessment Instrument (Mini-Cog) questionnaire, a tool for the rapid identification of possible cognitive impairment in older individuals [31], was used to screen individuals with potential cognitive impairment. The Mini-Cog consists of a three-word recall test and a clock drawing test with a total score of 5 points. In the three-word recall test, 1 point was scored for every correct word that was successfully recalled. In the clock drawing test, a normal clock with all numbers placed in the correct sequence and position and two hands pointing to the right numbers were scored for two points. Individuals with a total score < 3 were considered to have MCI or dementia and were thus excluded from further SCD evaluation. The accuracy of the Mini-Cog for detecting cognitive impairment has been demonstrated in a meta-analysis with a pooled sensitivity of 0.91 (*95% CI*, 0.80–0.96) and a pooled specificity of 0.86 (*95% CI*, 0.74–0.93) [32].

The Chinese version of the 9-item Subjective Cognitive Decline Questionnaire-9 (SCD-Q9) was used to measure SCD symptoms. The SCD-Q9 is a simple and quick screening tool for SCD identification in the general population [33]. The SCD-Q9 contains two dimensions covering the nine core symptoms of SCD, overall memory function and time comparison (four items) and daily activity ability (five items), with each item scored 0 for ‘no’, 0.5 for ‘occasionally’, and 1 for ‘yes’. The total SCD-Q9 score ranges from 0 to 9, with higher scores indicating worse SCD symptoms [33]. The Chinese version of the SCD-Q9 has good internal reliability and validity, and has been demonstrated to be suitable for screening SCD among older Chinese adults [34]. In this study, the Cronbach’s alpha was 0.729. A cutoff point of 5 was used: a score of 5 or higher was defined as SCD, and a score of less than 5 was defined as non-SCD [33,35,36].

### 2.3. Dietary Data Collection and Assessment of Dietary Diversity

The Dietary Quality Questionnaire (DQQ) [28] was used to collect dietary data. The DQQ comprises yes/no questions about the consumption of sentinel foods corresponding to 29 food groups in the previous 24 h. The food groups are as follows: (1) foods made from grains; (2) whole grains; (3) white roots, tubers, and plantains; (4) legumes; (5) vitamin A-rich orange vegetables; (6) dark green leafy vegetables; (7) other vegetables; (8) vitamin A-rich fruits; (9) citrus; (10) other fruits; (11) baked/grain-based sweets; (12) other sweets; (13) eggs; (14) cheese; (15) yogurt; (16) processed meats; (17) unprocessed red meat (ruminant, for example, beef, lamb, and goat); (18) unprocessed red meat (nonruminant, for example, pork); (19) poultry; (20) fish and seafood; (21) nuts and seeds; (22) packaged ultraprocessed salty snacks; (23) instant noodles; (24) deep fried foods; (25) fluid milk; (26) sugar-sweetened beverages (soft drinks); (27) fruit juice and fruit-flavored drinks; (28) sweet tea/coffee/cacao; and (29) fast food. The reliability of the Chinese version of the DQQ has been verified for its ability to capture food group consumption in the Chinese population [37].

To assess dietary diversity, the Food Group Diversity Score (FGDS) and Consumed All Five Recommended Food Score (All-5) were calculated based on DQQ data [28]. The FGDS ranges from 0 to 10, by summing the scores for each of the following ten groups aggregated from 29 food groups of DQQ (0: not consumed; 1: consumed): (1) grains, white roots and tubers, and plantains; (2) pulses (beans, peas, and lentils); (3) nuts and seeds; (4) dairy; (5) meat, poultry, and fish; (6) eggs; (7) dark green leafy vegetables; (8) other vitamin A-rich fruits and vegetables; (9) other vegetables; and (10) other fruits. The FGDS reflects food group diversity, with a score < 5 indicating inadequate micronutrient intake and a higher score indicating more adequate intake. The All-5 score reflects the adequacy of the five food groups recommended by most dietary guidelines worldwide by summing the scores for each of the following five groups aggregated from 29 food groups of the DQQ (0: not consumed; 1: consumed): (1) starchy staples, (2) vegetables, (3) fruits, (4) pulses, nuts, and seeds; and (5) animal-source foods. The All-5 score ranges from 0 to 5, with a score of 5 indicating minimal adherence to dietary guidelines, and each missing point indicating one food group missed in the daily diet.

### 2.4. Assessment of Covariates

The following variables were included in the confounding adjustments: social demographic information including sex, age, marital status (married, widowed, and other), educational level (primary school and lower, middle school, high school, and higher education), and annual family income (less than CNY 50,000, CNY 50,000–100,000, CNY 100,000–200,000, and more than CNY 200,000); lifestyle behavioral information including physical activity, smoking (non-smoker, current, and former smoker), and alcohol drinking (non-drinker, moderate, and heavy drinker); and health- and medical-related information including body mass index (BMI), self-reported weight change in the past year, medical history, and sleep quality. Data were collected using questionnaires. BMI was calculated based on self-reported height and weight. Self-reported weight changes in the past year were categorized into three categories: significant weight decline (weight loss of more than 5% of current body weight), significant weight gain (weight increase of more than 5% of current body weight), and no significant change (weight change between −5% and 5% of current body weight). History of hypertension, hyperlipidemia, diabetes, heart disease, arthritis, cancer, and other chronic diseases was collected based on self-reported information, and the number of comorbidities was counted based on self-reported medical history. The Physical Activity Rating Scale-3 (PARS-3) was used to assess physical activity levels (low, ≤19; moderate, 20–42; and high, ≥43) [38]. Sleep quality was assessed using the Pittsburgh Sleep Quality Index (PSQI) and categorized as good (PSQI score ≤ 5) or poor (PSQI score > 5) [39].

### 2.5. Statistical Analysis

Data are expressed as counts (%) for categorical variables and mean ± standard deviation (sd) for continuous variables. FGDS and All-5 scores were expressed as continuous variables or converted into different categories according to their distributions and implications: for FGDS, ≤4 (inadequate micronutrient intake), 5–7 (minimal adequate intake), and ≥8 (better adequate intake), and for All-5, 5 (minimal adherence to dietary guideline), 4 (missing one food group in daily diet), and ≤3 (missing two or more than two food groups in daily diet). The Chi-square test, *t*-test, or Mann–Whitney U test was used to compare the differences in characteristics between the SCD and non-SCD groups. Multivariable logistic regression analyses were performed to evaluate the association of FGDS and All-5 scores (as continuous or categorical variables) with SCD risk, and odds ratios (*OR*s) with 95% confidence intervals (*CIs*) were calculated. Three models were constructed: Model 1 was adjusted for age and sex; Model 2 was further adjusted for marital status, educational level, and total annual family income; and Model 3 was additionally adjusted for physical activity level, BMI, smoking, alcohol consumption, sleep quality, self-reported weight change in the past year, and the number of chronic comorbidities. The interaction effects between the All-5 score as a categorical variable and demographic or lifestyle factors were analyzed using fully adjusted logistic regression models. Subgroup analyses were performed to examine whether demographic or lifestyle factors modulated the association between All-5 score and SCD risk. For sensitivity analysis, cutoff points of 3 and 6 on the SCD-Q9 were further used to define SCD, and multivariable logistic regression analyses with full adjustment for covariates were performed to examine the associations of FGDS and All-5 scores with SCD risk. Statistical significance was defined as a two-sided *p*-value < 0.05. All statistical analyses were performed using SPSS version 26.0 (IBM Corporation, New York, NY, USA).

## 3. Results

Among the 946 eligible participants, 75 had potential cognitive impairment based on the Mini-Cog assessment (score < 3) and were excluded from further analysis. Compared with those without cognitive impairment, these participants were older, less educated, and had a lower family income (Table S1). Therefore, 871 participants without potential cognitive impairment were included in the final analysis. The mean age of the participants was 70.8 ± 8.3 years. Among them, 525 (60.3%) were women, and 80.5% were aged 65 years and older (Table 1). In total, 358 (41.1%) were classified as SCD and 513 (58.9%) as non-SCD. The mean SCD-Q9 scores of the participants in the SCD and non-SCD groups were 5.9 ± 0.9 and 2.6 ± 1.4, respectively. Participants in the SCD group were more likely to be women, were less educated, had a higher number of comorbidities, and had a higher proportion of hyperlipidemia and arthritis. In addition, more participants in the SCD group reported worse sleep quality and a significant decrease in body weight over the previous year (Table 1). There were no significant differences in age, marital status, family income, physical activity level, smoking, alcohol consumption, or other chronic medical conditions between the SCD and non-SCD groups (Table 1).

The FGDSs of all participants ranged from 2 to 10, with an average of 6.0 ± 1.5. Among all participants, 16.8% might have inadequate micronutrient intake (FGDS ≤ 4), 66.7% might have minimal adequate micronutrient intake (5 < FGDS ≤ 7), and 16.5% were classified as having better micronutrient intake (FGDS ≥ 8) (Table 2). There was no significant difference in the average FGDSs between the SCD and non-SCD groups (non-SCD, 6.1 ± 1.5 vs. SCD, 5.9 ± 1.5; *p* = 0.06). However, there was a higher proportion of participants in the low-FGDS range (≤4) and a lower proportion in the high-FGDS range (≥8) in the SCD group than in the non-SCD group (*p* = 0.04) (Table 2). No differences in the consumption of individual FGDS food groups were observed between the non-SCD and SCD groups (Table S2). The All-5 scores of all the participants ranged from 2 to 5, with an average of 4.2 ± 0.7. Among all participants, 35.1% had minimal adherence to dietary guidelines (All-5 = 5), and there were 49.8% and 15.0% participants missing one (All-5 = 4) and more than one (All-5 ≤ 3) food group in daily diet, respectively (Table 2). Participants in the SCD group had significantly lower scores than those in the non-SCD group (SCD, 4.1 ± 0.7 vs. non-SCD, 4.2 ± 0.7; *p* < 0.01, independent-sample Mann–Whitney U test). Similarly, there were more participants who scored less than 4 and fewer participants who scored 5 in the SCD group than in the non-SCD group (*p* = 0.02) (Table 2). In addition, participants in the non-SCD group (84.8%) had a higher proportion of fruit consumption than those in the SCD group (78.8%) (*p* = 0.02), although the consumption of other All-5 food groups was not different (Table S2).

Multivariate logistic regression analysis was used to analyze the association between the dietary diversity scores and SCD risk (Table 3). In the age- and sex-adjusted model (Model 1) and the model further adjusted for other sociodemographic factors (Model 2), higher FGDS and All-5 scores were significantly associated with a lower SCD risk. In the fully adjusted model (Model 3, additionally adjusted for lifestyle, health, and disease-related factors), the association between FGDS and SCD risk became insignificant (*OR* = 0.92, *95% CI*: 0.83–1.01; *p* = 0.08); however, the association between All-5 score and SCD risk (*OR* = 0.79, *95% CI*: 0.65–0.97; *p* = 0.02) remained significant. Compared with participants with the highest FGDS (≥8), those with the lowest FGDS (≤4) had an 85% higher risk of SCD (*OR* = 1.85, *95% CI:* 1.10–3.13; *p* = 0.02) after adjusting for all covariates. Similarly, compared with participants with an All-5 score of 5, those with an All-5 score of 3 or lower had a 90% increased SCD risk (*OR* = 1.90, *95% CI*: 1.21–2.97; *p* < 0.01).

When analyzing the individual food groups of the All-5 components, fruits were the only food group significantly associated with SCD risk (Table S3). The effect of fruit consumption on SCD risk was further analyzed (Table 4). Compared with participants who consumed all All-5 food groups (All-5 = 5), those who did not consume fruits (All-5 < 5 without fruits) had a significantly increased SCD risk (*OR* = 1.63, *95% CI:* 1.06–2.50; *p* = 0.03), whereas SCD risk did not change in participants whose All-5 food groups included fruits (All-5 < 5 with fruits) (*OR* = 1.17, *95% CI:* 0.85–1.612; *p* = 0.34).

**Figure 1:**
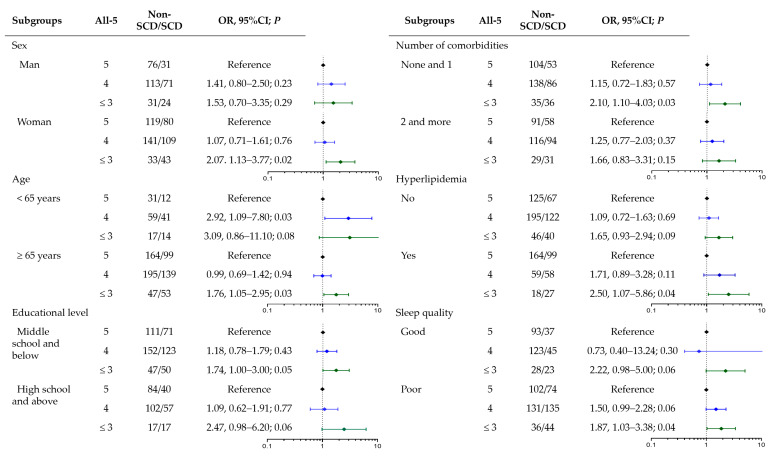
Association of All-5 scores with SCD stratified by SCD-related risk factors.

Educational level, sleep quality, and the number of comorbidities were factors with different distributions between SCD and non-SCD participants (Table 1). No interaction effect was found between the FGDS or All-5 scores and these covariates. Subgroup analyses were performed to examine the association between the All-5 score and SCD risk in the different populations (Figure 1). Similar trends of increased SCD risk by lower All-5 scores were observed in all subgroups, but the associations were significant only in women, those aged ≥ 65 years, with lower educational level, without multiple chronic medical conditions, and with poor sleep quality.

Sensitivity analyses were conducted by regressing diversity scores in SCD classified with different cutoff points on the SCD-Q9 scores. At the cutoff point of 3, more SCD individuals were identified (555 of 871, 63.7%), and neither FGDS nor All-5 was significantly associated with SCD risk (Table 5). Much fewer SCD individuals were identified at the cutoff point of 6 (115 out of 871, 13.2%), and logistic regression results showed that individuals in the lowest FGDS group (*OR* = 2.29, *95% CI:* 1.03–5.09; *p* = 0.04) and the lowest All-5 group (*OR* = 2.08, *95% CI*: 1.14–3.80; *p* = 0.02) had significantly increased SCD risks, which were comparable with those found at the cutoff point of 5 (Table 5).

## 4. Discussion

In this cross-sectional study among middle-aged and elderly residents, we found that higher FGDS and All-5 scores, two indicators of dietary diversity derived from the DQQ, were associated with a lower risk of SCD; particularly, we showed that fruits were the major food group that was significantly related to SCD risk. Our results underscore the critical role of a balanced diet in delaying age-related cognitive decline and emphasize the importance of regular fruit consumption.

Few studies have investigated the relationship between dietary diversity and SCD risk. In our study among middle-aged and elderly participants, we found that individuals with the lowest FGDSs, an indicator of food group diversity, had an 85% increase in SCD risk. Our study is consistent with a previous study reporting that a higher dietary diversity score derived from dietary recall data was associated with a 15% decrease in the risk of self-reported memory decline, an important symptom of SCD, in Chinese participants aged 50 years and older [26]. Our results are also in accordance with those of other studies that investigated the effects of dietary quality on SCD [15,16,17]. For example, among participants of the Health Professionals Follow-up Study and the Nurses’ Health Study, greater adherence to the Alternate Mediterranean Diet (AMED) was shown to decrease the risk of developing severe SCD by 43% [15]. In another study, mid-life adherence to the Dietary Approaches to Stop Hypertension (DASH) diet was associated with a 17% decrease in late-life subjective cognitive complaints in women [17].

The negative association between FGDS and SCD risk was further supported by the results showing that a lower All-5 score, an indicator of adherence to dietary guideline recommendations, was associated with an increased SCD risk. All-5 measures the intake of starchy staples, vegetables, fruits, pulses/nuts/seeds, and animal-source foods, five food groups whose daily consumption is recommended by most dietary guidelines worldwide [18,19,20]. A score of 5 indicates consumption of all five food groups and minimal adherence to dietary guidelines, and a score of less than 5 indicates that certain food groups are missed from a daily diet. In this study, we found a negative association between All-5 score and SCD risk, with a 90% higher risk of SCD in individuals with at least two food groups missed in the daily diet. The finding that both FGDS and All-5 scores were negatively related to SCD risk highlights the critical role of unbalanced diets or poor dietary quality in the development of SCD, which has been demonstrated in previous studies [15,16,17,26].

Among the All-5 food groups, we further found that fruits contributed most significantly to SCD risk, as a diet without fruits was related to an approximately 63% increase in SCD risk. The finding is consistent with a previous report that higher intake of fruits and vegetables was associated with better subjective cognitive function in later life among adult men in the US [40]. The health benefits of fruits and vegetables are well recognized [41], and protective effects of fruits and vegetables against cognitive impairment and dementia have been reported in numerous studies [40,42,43]. These protective effects may be attributed to antioxidative compounds rich in fruits and vegetables, such as vitamin C, carotenoids, and flavonoids. These compounds have been shown to alleviate oxidative stress, reduce inflammation, and protect against neuronal damage, thereby delaying cognitive decline [44]. In support of this, studies have observed a decreased risk of SCD among populations with a long-term high intake of carotenoids [13] and flavonoids [14].

In this study, we used DQQ-derived FGDS and All-5 scores as indicators of dietary diversity. By collecting the intake of sentinel foods in a 24 h period, the DQQ was originally designed as a low-cost and convenient tool for the quick assessment of dietary quality at the population level. Studies have shown that sentinel foods selected in the China-adapted DQQ account for over 95% of people who consume any item in each food group [37], and DQQ-derived dietary diversity scores and dietary diversity scores derived from a 3-day recall have been shown to be comparably associated with psychological stress [29]. In this study, we found that DQQ-derived diversity indicators were negatively linked to cognitive decline, similar to the associations between other well-established diversity scores and cognitive function [25,26,45]. These findings suggest the sound representativeness and accuracy of DQQ-derived dietary diversity indicators and endorse their potential application in relating to health outcomes. However, the DQQ focuses on food group intake and does not quantitatively collect intake information for a specific food, which limits its application in detailed dietary analyses. In this study, we were not able to identify specific fruits that might be related to SCD or examine the relationship between vegetable intake and SCD risk because more than 95% of the study population had daily vegetable consumption. Dietary assessment using a dietary recall method or food frequency questionnaire is needed to further investigate the relationship between dietary intake and SCD.

SCD is a state of self-perceived cognitive decline before impairment becomes detectable on standardized neuropsychological tests and has been recognized as an early symptomatic expression of preclinical dementia [2]. In this study, we used the SCD-Q9 questionnaire to screen individuals with SCD symptoms and the Mini-Cog to exclude those with potential cognitive impairment, and a cutoff point of 5 was used for SCD classification. We found that poor dietary diversity increased SCD risk, and this negative association was independent of age, sex, educational level, sleep quality, and presence of chronic diseases. Our findings highlight the importance of dietary quality in maintaining normal cognitive function and suggest a potential role of adapting a balanced diet and following dietary guideline recommendations for the early prevention of dementia diseases. However, it should be noted that in addition to being an early manifestation of dementia, SCD is also related to other etiologies, such as poor sleep quality [46,47], mental disorders such as depression and anxiety [47], and presentation of chronic medical conditions [4]. A cross-sectional association between dietary quality and SCD caused by factors other than dementia pathology could not be excluded. A longitudinal study is warranted to further investigate the effects of dietary quality on the transition from subjective to objective cognitive impairment.

To the best of our knowledge, this is the first study to examine the association between dietary diversity and SCD in a community-dwelling Chinese population. However, this study has several limitations. First, owing to its cross-sectional design, the study revealed an association at a specific time point. SCD is a process of cognitive decline that can remit fully, remain stable but irreversible, or transition to cognitive impairment and dementia [2]. How a diet with better diversity affects SCD transitions remains unknown. Furthermore, the possibility of reverse causality, in which SCD affects dietary quality, cannot be excluded. Second, as a quick assessment tool, the DQQ collects the types, but not the quantity, of food intake in a 24 h period, which makes it impossible to determine the effect of specific food or the dose–response effect of food consumed by most people (i.e., the effect of vegetable intake on SCD). Third, because of the lack of standardization of the SCD-Q9, different cutoff points have been applied in many studies with different sensitivities and specificities [36,48,49]. In this study, we found that the association was attenuated or strengthened when a cutoff point other than five was used. Finally, there is an issue with the generalization of the study results. The participants in our study were from a community located in eastern China, and their dietary habits, education levels, economic conditions, and other demographic characteristics could not represent populations in other regions of China. Despite these limitations, our preliminary study provides important clues regarding the relationship between dietary diversity and SCD in Chinese adults. Further investigation adopting a longitudinal design and a more sophisticated dietary assessment tool (such as dietary recall or food frequency questionnaires) is warranted.

## 5. Conclusions

In conclusion, our study showed that poor dietary diversity was associated with a higher risk of SCD in middle-aged and older adults, with fruit being the food group with the most significant effect. Our findings highlight the importance of adapting a balanced diet and following dietary guideline recommendations for maintaining cognitive health, which has practical implications in nutritional education and interventions to prevent age-related cognitive diseases and promote elderly health.

## Figures and Tables

**Table 1:** Demographic characteristics of total participants and stratified by SCD status ^a^.

Variables		Total (*n* = 871)	SCD		
			No (*n* = 513)	Yes (*n* = 358)	*p* ^b^
SCD Score		4.0 (2.0)	2.6 (1.4)	5.9 (0.9)	
Age, yrs		70.8 (8.3)	70.5 (8.3)	71.2 (8.3)	0.26
Sex, *n*(%)	Man	346 (39.7)	220 (42.9)	126 (35.2)	0.02 *
	Woman	525 (60.3)	293 (57.1)	232 (64.8)	
Marital Status, *n*(%)	Married	723 (83.0)	422 (82.3)	301 (84.1)	0.77
	Widowed	137 (15.7)	84 (16.4)	53 (14.8)	
	Other	11 (1.3)	7 (1.4)	4 (1.1)	
Educational level, *n*(%)	Primary school and Lower	198 (22.7)	102 (19.9)	96 (26.8)	0.03 *
	Middle school	356 (40.9)	208 (40.5)	148 (41.3)	
	High school	198 (22.7)	122 (23.8)	76 (21.2)	
	Higher education	119 (13.7)	81 (15.8)	38 (10.6)	
Family Income, *n*(%)	Less than CNY 50,000	108 (12.4)	58 (11.3)	50 (14.0)	0.17
	CNY 50,000–100,000	288 (33.1)	170 (33.1)	118 (33.0)	
	CNY 100,000–200,000	396 (45.5)	230 (44.8)	166 (46.4)	
	Higher than CNY 200,000	79 (9.1)	55 (10.7)	24 (6.7)	
BMI, kg/m^2^		23.5 (3.3)	23.6 (3.3)	23.2 (3.4)	0.06
Weight Change in the past year, *n*(%)	No change	709 (81.4)	435 (84.8)	274 (76.5)	<0.01 **
	Significant Decrease	114 (13.1)	52 (10.1)	62 (17.3)	
	Significant Increase	48 (5.5)	26 (5.1)	22 (6.1)	
Physical Activity level, *n*(%)	Low	509 (58.4)	295 (57.5)	214 (59.8)	0.62
	Moderate	277 (31.8)	164 (32.0)	113 (31.6)	
	High	85 (9.8)	54 (10.5)	31 (8.7)	
Smoking, *n*(%)	Non-Smoker	642 (73.7)	370 (72.1)	272 (76.0)	0.21
	Current smoker	108 (12.4)	72 (14.0)	36 (10.1)	
	Former smoker	121 (13.9)	71 (13.8)	50 (14.0)	
Alcohol Drinking, *n*(%)	Non-Drinker	658 (75.5)	386 (75.2)	272 (76.0)	0.09
	Moderate Drinker	135 (15.5)	73 (14.2)	62 (17.3)	
	Heavy Drinker	78 (9.0)	54 (10.5)	24 (6.7)	
Sleep Quality, *n*(%)	Good	349 (40.1)	244 (47.6)	105 (29.3)	<0.001 **
	Poor	522 (59.9)	269 (52.4)	253 (70.7)	
Hypertension, *n*(%)	Yes	538 (61.8)	325 (63.4)	213 (59.5)	0.25
Diabetes, *n*(%)	Yes	220 (25.3)	125 (24.4)	95 (26.5)	0.47
Hyperlipidemia, *n*(%)	Yes	276 (31.7)	147 (28.7)	129 (36.0)	0.02 *
Heart disease, *n*(%)	Yes	109 (12.5)	57 (11.1)	52 (14.5)	0.13
Gout, *n*(%)	Yes	49 (5.6)	29 (5.7)	20 (5.6)	0.97
Chronic kidney disease, *n*(%)	Yes	12 (1.4)	7 (1.4)	5 (1.4)	0.97
Cancer, *n*(%)	Yes	50 (5.7)	29 (5.7)	21 (5.9)	0.89
Chronic lung disease, *n*(%)	Yes	23 (2.6)	11 (2.1)	12 (3.4)	0.27
Hearing Disorder, *n*(%)	Yes	18 (2.1)	7 (1.4)	11 (3.1)	0.08
Arthritis, *n*(%)	Yes	94 (10.8)	33 (6.4)	61 (17.0)	<0.001 **
Number of comorbidity, *n*(%)	0	153 (17.6)	94 (18.3)	59 (16.5)	<0.01 **
	1	299 (34.3)	183 (35.7)	116 (32.4)	
	2	233 (26.8)	149 (29.0)	84 (23.5)	
	3	136 (15.6)	66 (12.9)	70 (19.6)	
	4 and more	50 (5.7)	21 (4.1)	29 (8.1)	

^a^ data are expressed as mean ± standard deviation (sd) for continuous variables and count (%) for categorical variables; ^b^ independent *t*-test or χ^2^ test; * *p* < 0.05; ** *p* < 0.01.

**Table 2:** Dietary diversity scores of total participants and stratified by SCD status ^a^.

	Total (*n* = 871)	SCD		
		No (*n* = 513)	Yes (*n* = 358)	*p*
FGDS, continuous	6.0 ± 1.5	6.1 ± 1.5	5.9 ± 1.5	0.06 ^b^
FGDS category				
≤4	146 (16.8)	79 (15.4)	67 (18.7)	0.04 ^c,^*
5–7	581 (66.7)	336 (65.5)	245 (68.4)	
≥8	144 (16.5)	98 (19.1)	46 (12.8)	
All-5 Score, continuous	4.2 ± 0.7	4.2 ± 0.7	4.1 ± 0.7	<0.01 ^d,^**
All-5 category				
≤3	131 (15.0)	64 (12.5)	67 (18.7)	0.02 ^c,^*
4	434 (49.8)	254 (49.5)	180 (50.3)	
5	306 (35.1)	195 (38.0)	111 (31.0)	

^a^ Data are expressed as mean ± sd for FGDS and All-5 scores, and count (%) for score levels; ^b^ *t*-test; ^c^ *χ^2^* test; ^d^ Mann–Whitney U test; * *p* < 0.05; ** *p* < 0.01.

**Table 3:** Odds ratio (*OR*) and 95% confidential interval (*CI*) of SCD across dietary diversity scores among middle-aged and elderly participants (*OR*, *95% CI*; *p*) ^a^.

	Non-SCD/SCD (513/358)	Model 1	Model 2	Model 3
FGDS, continuous		0.90, 0.82–0.98; 0.02 *	0.91, 0.83–0.99; 0.04 *	0.92, 0.83–1.01; 0.08
FGDS category				
≥8	98/46	Reference	Reference	Reference
5–7	336/245	1.61, 1.09–2.37; 0.02 *	1.54, 1.04–2.29; 0.03 *	1.48, 0.98–2.22; 0.06
≤4	79/67	1.95, 1.20–3.17; <0.01 **	1.81, 1.10–2.99; 0.02 *	1.85, 1.10–3.13; 0.02 *
All-5 Score, continuous		0.76, 0.63–0.91; <0.01 **	0.77, 0.64–0.94; <0.01 **	0.79, 0.65–0.97; 0.02 *
All-5 category				
5	195/111	Reference	Reference	Reference
4	254/180	1.29, 0.95–1.75; 0.10	1.27, 0.93–1.73; 0.13	1.2, 0.87–1.66; 0.26
≤3	64/67	1.91, 1.26–2.89; <0.01 **	1.83, 1.19–2.81; <0.01 **	1.90, 1.21–2.97; <0.01 **

^a^ Model 1 adjusted for age and gender; Model 2 further adjusted for marital status, educational level, and total annual family income; Model 3 additionally adjusted for BMI, physical activity, smoking, alcohol consumption, sleep quality, self-reported weight change in the past year, and number of chronic comorbidities. * *p* < 0.05; ** *p* < 0.01.

**Table 4:** Odds ratio (*OR*) and 95% confidential interval (*CI*) of SCD across participants with or without fruit consumption (*OR*, *95% CI*; *p*) ^a^.

Fruit Consumption	Non-SCD/SCD	*OR*, *95% CI*; *p*
All-5 = 5	195/111	Reference
All-5 < 5 with fruits	240/171	1.17, 0.85–1.62; 0.35
All-5 < 5 without fruits	78/76	1.63, 1.07–2.50; 0.03 *

^a^ Adjusted for age, gender, marital status, educational level, total annual family income, BMI, physical activity, smoking, alcohol consumption, sleep quality, self-reported weight change in the past year, and number of chronic comorbidities. * *p* < 0.05.

**Table 5:** Odds ratio (*OR*) and 95% confidential interval (*CI*) of SCD classified by different cutoff points across dietary diversity scores (*OR*, *95% CI*; *p*) ^a^.

	Cutoff Point at 3		Cutoff Point at 6	
	Non-SCD/SCD	*OR*, *95% CI*; *p*	Non-SCD/SCD	*OR*, *95% CI*; *p*
	(316/555)		(756/115)	
FGDS, continuous		0.93, 0.85–1.03; 0.18		0.89, 0.77–1.03; 0.12
FGDS category				
≥8	57/87	Reference	133/11	Reference
5–7	210/371	1.11, 0.74–1.66; 0.61	503/78	1.67, 0.84–3.32; 0.14
≤4	49/97	1.29, 0.76–2.18; 0.35	120/26	2.29, 1.03–5.09; 0.04 *
All-5 Score, continuous		0.89, 0.72–1.10; 0.29		0.75, 0.57–1.00; 0.05 *
All-5 category				
5	115/191	Reference	274/32	Reference
4	162/272	0.96, 0.69–1.33; 0.80	379/55	1.14, 0.70–1.86; 0.59
≤3	39/92	1.42, 0.88–2.29; 0.15	103/28	2.08, 1.14–3.80; 0.02 *

^a^ Adjusted for age, gender, marital status, educational level, total annual family income, BMI, physical activity, smoking, alcohol consumption, sleep quality, self-reported weight change in the past year, and number of chronic comorbidities. * *p* < 0.05.

## Data Availability

Data will be made available on request.

## Supplementary Materials

The following supporting information can be downloaded at https://www.mdpi.com/article/10.3390/nu16213603/s1: Table S1: Comparison of characteristics between participants with and without cognitive impairment; Table S2: Food groups consumed by all participants and by SCD status; Table S3: Odds ratio (*OR*) and 95% confidential interval (*CI*) of SCD across All-5 food groups among middle-aged and elderly participants (*OR, 95% CI*; *p*).
