# Supplementary material for: Association Between Dietary Diversity and Subjective Cognitive Decline in the Middle-Aged and Elderly Chinese Population: A Cross-Sectional Study
Source: Nutrients. 2024 Oct 23;16(21):3603. doi: 10.3390/nu16213603 (PMC11548035; doi:10.3390/nu16213603)
Supplement: Supplementary file 1 [file nutrients-16-03603-s001.zip › nutrients-3224564-supplementary.pdf]

Table S1. Comparison of characteristics between participants with and without cognitive impairment

Variables		No-Cognitive Impairment (n = 871)	Cognitive Impairment (n = 75)	*P*
Mini-Cog score		4.2 ± 0.8	1.2 ± 0.8	< 0.001*
SEX, n(%)	Man	346 (39.7)	23 (30.7)	0.12
	Woman	525 (60.3)	52 (69.3)	
Age, yrs		70.8 ± 8.3	73.4 ± 9.4	0.01*
Educational level, n (%)	Primary school and Lower	198 (22.7)	39 (52.0)	< 0.001*
	Middle school	356 (40.9)	23 (30.7)	
	High school	198 (22.7)	7 (9.3)	
	Higher education	119 (13.7)	6 (8.0)	
Family income, n (%)	Less than 50,000 CNY	108 (12.4)	12 (16.0)	0.02*
	50,000 - 100,000 CNY	288 (33.1)	36 (48.0)	
	100,000 - 200,000 CNY	396 (45.5)	23 (30.7)	
	Higher than 200,000 CNY	79 (9.1)	4 (5.3)	
Martial status, n (%)	Married	723 (83.0)	57 (76.0)	0.29
	Widowed	137 (15.7)	17 (22.7)	
	Other	11 (1.3)	1 (1.3)	
BMI, Kg/m^2		23.5 ± 3.3	22.8 ± 3.0	0.08
Physical Activity level, n (%)	Low	509 (58.4)	47 (62.7)	0.63
	Moderate	277 (31.8)	23 (30.7)	
	High	85 (9.8)	5 (6.7)	
Smoking, n (%)	Non-Smoker	642 (73.7)	61 (81.3)	0.34
	Current smoker	108 (12.4)	7 (9.3)	
	Former smoker	121 (13.9)	7 (9.3)	
Alcohol Drinking, n (%)	Non-Drinker	658 (75.5)	62 (82.7)	0.38
	Moderate Drinker	135 (15.5)	8 (10.7)	
	Heavy Drinker	78 (9.0)	5 (6.7)	
Sleep Quality, n (%)	Good	349 (40.1)	28 (37.3)	0.64
	Poor	522 (59.9)	47 (62.7)	
Hypertension, n (%)	Yes	538 (61.8)	52 (69.3)	0.19
Diabetes, n (%)	Yes	220 (25.3)	21 (28.0)	0.6
Hyperlipidemia, n (%)	Yes	276 (31.7)	18 (24.0)	0.17
Coronary heart disease, n (%)	Yes	109 (12.5)	11 (14.7)	0.59
Gout, n (%)	Yes	49 (5.6)	5 (6.7)	0.71
Chronic kidney disease, n (%)	Yes	12 (1.4)	1 (1.3)	0.98
Cancer, n (%)	Yes	50 (5.7)	4 (5.3)	0.88
Chronic Lung Disease, n (%)	Yes	23 (2.6)	1 (1.3)	0.49
Hearing Loss, n (%)	Yes	18 (2.1)	1 (1.3)	0.66
Arthritis, n (%)	Yes	94 (10.8)	7 (9.3)	0.70
FGDS score		6.0 ± 1.5	5.3 ± 1.5	< 0.001*
All-5 score		4.2 ± 0.7	3.9 ± 0.7	< 0.001*

Table S2. Food groups consumed by all participants and by SCD status [a]

	Total	SCD		
	(n = 871)	No (n = 513)	Yes (n = 358)	*P* [b]
FGDS food groups				
Grains	866 (99.4)	512 (99.8)	354 (98.9)	0.08
Pulses	237 (27.2)	150 (29.2)	87 (24.3)	0.11
Nuts and seeds	215 (24.7)	136 (26.5)	79 (22.1)	0.14
Dairy	423 (48.6)	255 (49.7)	168 (46.9)	0.42
Meat, poultry and fish	771 (88.5)	461 (89.9)	310 (86.6)	0.14
Eggs	609 (69.9)	359 (70.0)	250 (69.8)	0.96
Dark green vegetables	481 (55.2)	276 (53.8)	205 (57.3)	0.31
Vitamin A rich fruits and vegetables	229 (26.3)	138 (26.9)	91 (25.4)	0.63
Other vegetables	700 (80.4)	418 (81.5)	282 (78.8)	0.32
Other fruits	685 (76.8)	410 (78.2)	275 (74/7)	0.22
All-5 food groups				
Starch staples	866 (99.4)	512 (99.8)	354 (98.9)	0.08
Vegetables	836 (96.0)	493 (96.1)	343 (95.8)	0.83
Fruits	717 (82.3)	435 (84.8)	282 (78.8)	0.02*
Pulse, nuts, and seeds	376 (43.2)	233 (45.4)	143 (39.9)	0.11
Animal foods	849 (97.5)	501 (97.7)	348 (97.2)	0.67

[a] Data are expressed as n (%); [b] χ^2 test; * $P < 0.05$

Table S3. Odds ratio (OR) and 95% confidential interval (CI) of SCD across All-5 food groups among middle-aged and elderly participants (*OR, 95%CI; P*) [a]

Consumption of All-5 Food groups		non-SCD (n = 513)	SCD (n = 358)	*OR, 95%CI; P*
Starch staples	Yes	512	354	Reference
	No	1	4	6.17, 0.65 - 59.08; 0.11
Vegetables	Yes	493	343	Reference
	No	20	15	1.13, 0.55 - 2.34; 0.74
Fruits	Yes	435	282	Reference
	No	78	76	1.55, 1.06 - 2.26; 0.03*
Pulse, nuts, and seeds	Yes	233	143	Reference
	No	280	215	1.21, 0.90 - 1.62; 0.21
Animal foods	Yes	501	348	Reference
	No	12	10	1.23, 0.51 - 2.98; 0.65

[a] Adjusted for age, gender, marital status, educational level, total annual family income, BMI, physical activity, smoking, alcohol consumption, sleep quality, self-reported weight change in the past year, and number of chronic comorbidities. * $P < 0.05$.
